# Real-time and label free determination of ligand binding-kinetics to primary cancer tissue specimens; a novel tool for the assessment of biomarker targeting

**DOI:** 10.1016/j.sbsr.2016.05.003

**Published:** 2016-07

**Authors:** Thomas Mandel Clausen, Marina Ayres Pereira, Htoo Zarni Oo, Mafalda Resende, Tobias Gustavson, Yang Mao, Nobuo Sugiura, Janet Liew, Ladan Fazli, Thor G. Theander, Mads Daugaard, Ali Salanti

**Affiliations:** a Centre for Medical Parasitology at Department of Immunology and Microbiology, University of Copenhagen, Denmark; b Department of Infectious Diseases, Copenhagen University Hospital, Copenhagen, Denmark; c Vancouver Prostate Centre, Vancouver, BC V6H 3Z6, Canada; d Department of Urologic Sciences, University of British Columbia, Vancouver, BC, Canada; e Molecular Pathology and Cell Imaging Laboratory, Vancouver Prostate Centre, Vancouver, BC V6H 3Z6, Canada; f Copenhagen Center for Glycomics and Department of Cellular and Molecular Medicine, University of Copenhagen, Denmark; g Department of Biochemistry, Boston University School of Medicine, Boston, MA, USA; h Institute for Molecular Science of Medicine, Aichi Medical University, Japan

**Keywords:** Quartz crystal microscale, Biomarker, Biosensor, VAR2CSA, Cancer, Malaria

## Abstract

In clinical oncology, diagnosis and evaluation of optimal treatment strategies are mostly based on histopathological examination combined with immunohistochemical (IHC) expression analysis of cancer-associated antigens in formalin fixed paraffin-embedded (FFPE) tissue biopsies. However, informative IHC analysis depends on both the specificity and affinity of the binding reagent, which are inherently difficult to quantify *in situ*. Here we describe a label-free method that allows for the direct and real-time assessment of molecular binding kinetics *in situ* on FFPE tissue specimens using quartz crystal microbalance (QCM) enabled biosensor technology. We analysed the interaction between the rVAR2 protein and its placental-like chondroitin sulfate (pl-CS) receptor in primary human placenta tissue and in breast and prostate tumour specimens *in situ*. rVAR2 interacted with FFPE human placenta and cancer tissue with an affinity in the nanomolar range, and showed no detectable interaction with pl-CS negative normal tissue. We further validated the method by including analysis with the androgen receptor N-20 antibody (*anti*-AR). As the K_D_ value produced by this method is independent of the number of epitopes available, this readout offers a quantitative and unbiased readout for *in situ* binding-avidity and amount of binding epitopes. In summary, this method adds a new and important dimension to classical IHC-based molecular pathology by adding information about the binding characteristics in biologically relevant conditions. This can potentially be used to select optimal biologics for diagnostic and for therapeutic applications as well as guide the development of novel high affinity binding drugs.

## Highlights

• Proposes a method to analyze binding kinetics on FFPE tissue using QCM technology
• Adds to classic IHC by showing the binding characteristics of the targeting reagent
• Can be used to determine the relative presence of a cancer specific target
• rVAR2 interacts strongly with pl-CS in cancer and placental tissue only.
• A potential tool in drug development, patient stratification, and diagnostics

## 1 Introduction

Cancer is a capital health problem in the modern world and therefore a major focus of modern medical research. Currently, diagnosis and disease management of most types of cancer is based on histopathological examination, combined with a molecular pathology analysis of primary tumour and potential metastatic lesions [3]. Classical molecular pathology analysis involves IHC evaluation of biomarkers that can inform on disease status and cancer subtype, as well as on molecular signatures able to guide therapy. The standard analysis is two-dimensional in the sense that the output only informs on expression intensity and pattern of the biomarker(s) in question. Nevertheless, molecular pathology assessment plays an important role in the diagnosis and disease management of several types of cancer. For example, IHC staining intensity and pattern of ErbB2/HER2 is widely used to guide treatment in human breast cancer [4], [10], [30]. In prostate cancer, the expression intensity and pattern of the androgen receptor (AR) is used to predict the efficacy of *anti*-hormonal therapy [13]. However, the informative outcome of any molecular pathology analysis depends solely on the quality of the binding reagent and the avidity of the ligand-epitope interaction in *in situ*, which constitutes an inherent limitation of the method. Investigating the molecular characteristics of the interaction of a given targeting reagent with a biomarker could add extra information that would aid in disease diagnosis, patient stratification, as well as in evaluation for therapy [11].

Adding to the limited repertoire of informative tumour biomarkers, we have recently described a distinct chondroitin sulfate (CS) glycosaminoglycan (GAG) modification shared between the placental and malignant tissue compartments [27]. The observation was made in our work with placental malaria, in which infected erythrocytes express the VAR2CSA protein that allows them to anchor very specifically to a distinct type of CS in the placenta, thereby avoiding immune surveillance in the spleen [6], [7], [28]. Interestingly this distinct type of placental-like CS (pl-CS) is present on most cancer cells as well, while absent from normal tissue except placenta. This demonstrates the status of pl-CS as a cancer specific oncofetal antigen. pl-CS can be detected *in vitro*, *in vivo* and *in situ* using recombinant VAR2CSA proteins (rVAR2) [27]. The pl-CS modification is broadly present across multiple tumour types and the intensity of pl-CS tissue staining correlates with progression of malignant melanoma and predicts poor recurrence-free survival in non-small cell lung cancer patients [27]. Although a promising new cancer biomarker detection-reagent, rVAR2 is subject to the same limitations of standard molecular pathology analysis as any other established biomarker reagent, lacking important information on ligand-epitope binding avidity.

The Attana biosensor is an acoustic biosensor that measures changes in mass using the piezoelectric capacity of a quartz crystal (QCM technology) [17]. The change in mass per unit area on the crystal is directly proportional to the change in the crystals resonant frequency [29]. This allows for the use of the QCM technology as a microscale to measure small changes in mass, such as binding of an analyte to its immobilized ligand. More importantly, unlike surface plasmon resonance (SPR, Biacore [25], [33]), which relies on the reflection of polarized light, the QCM platform is independent of the composition of the immobilized ligand. This technology therefore allows for the characterization of the binding and kinetic properties of a given analyte's interaction with a ligand in its native environment using fixed cells [18], [23], [24], and recently also live cells [16]. However, to date this has not been attempted for *in situ* FFPE tissue analysis of primary patient biopsies. Here, we present a method that adds a third dimension to classical two-dimensional molecular pathology assessment by incorporating a kinetic readout of analyte-ligand interactions *in situ* using QCM biosensor technology.

## 2 Materials and methods

### 2.1 Materials and reagents

Antibodies used for immunohistochemistry (IHC) and immunofluorescence (IF) were *anti*-AR rabbit polyclonal antibody (1:200, N-20, Santa Cruz, sc-816), *anti*-V5-FITC antibody (1:500, Life Technologies, 46–0308) and fluorescein *anti*-rabbit IgG antibody (1:500, Vector Laboratories, FI-1000).

### 2.2 Immunohistochemistry

Tissue stainings were performed using the Ventana Discovery platform. Sectioned paraffin embedded tissue samples were deparaffinized in EZ prep solution (Ventana) and stained with V5-tagged rVAR2 or *anti*-AR (N-20, Santa Cruz) antibody. In brief, tissue sections were incubated in Cell Conditioning 1 (CC1) or Cell Conditioning 2 (CC2) solution (Ventana) to retrieve antigen, followed by incubation with primary staining molecules. Bound rVAR2 was detected with an *anti*-V5 antibody, and an anti-mouse-HRP detection step. Bound antibodies were incubated with universal secondary antibody and visualized using Streptavidin-biotin peroxidase detection system and 3.3′-diaminobenzidine as chromogen.

### 2.3 Tissue immobilization

Attana COP-1 cartridges were disassembled to retrieve the COP-1 gold chip. The chip was coated in poly-l-lysine solution 5min at RT (Sigma) prior to tissue immobilization. The chip was rinsed in PBST and allowed to dry at RT for 2h. The FFPE tissue, tested positive for the given target in IHC, was macro-dissected at 5μm thickness on a microtome. The tissue was mounted on the chip and baked at 60°C for 1h.

### 2.4 Chip deparaffinization and antigen retrieval

The tissue chips were deparaffinized in EZ prep solution at 65°C in a water bath for 30min, and rinsed three times in PBS. Antigen retrieval was performed in 10mM sodium citrate, 0.05% Tween, pH 6 for 30min at 95°C. The chip was again rinsed three times in PBS. For AR experiments, the tissue was permeabilized in 0.5% saponin in PBS 10min at RT. For antibody experiments the chips were blocked in 1% BSA, 10% FBS in PBS for 1 h at RT. For rVAR2 experiments, the chips were blocked in Synblock (Immunochemistry Technologies) solution for 1h at RT. The treated tissue chips were mounted in the COP-1 cartridges, being very careful not to allow leakage of fluid to under the chip. In the optimization step the tissue was stained with ethidium bromide, and the integrity of the tissue was viewed in a top-down fluorescence microscope.

### 2.5 Cell chip preparation

Cells were seeded on COP-1 surfaces (Attana AB) at 80.000 cells in 700 uL of appropriate supplemented growth media. Following a 24h incubation at 37°C, 5% CO_2_, the cells were fixed 10min in 4% PFA in PBS. The cells were washed in PBS and the cartridge was assembled, being careful of leakage. In the optimization step the cells were stained with ethidium bromide, and viewed in a top-down fluorescence microscope.

### 2.6 Purification of pl-CS from human placenta

5g of frozen placental tissue was minced and extensively washed with cold PBS. The tissue was then treated with 10mg collagenase type IV (Life Technologies) in 10mL PBS containing Ca^2 +^ and Mg^2 +^, for 16h at 37°C. The resulting supernatant was cleared at 3000g for 10min. The pellet was further digested with Trypsin EDTA (Lonza) for 2h at 37°C. The resulting supernatant was cleared at 3000g for 10min. All the supernatants were combined and lyophilized. The dried extract was delipidated using a series of chloroform and methanol washes using 2:1, 1:1, 1:2 *v*/v ratios and dried at 55°C. The sample was then digested with 20mg Pronase (Roche) in 20mL 50mM Tris/HCl, pH7 and 5mM CaCl_2_ for 16h at 50°C. After heat inactivation of the Pronase, 2mM MgCl_2_ and 1000U Benzonase (Sigma-Aldrich) were added and the sample was incubated for 2h at 37°C. The sample was run on a DEAE column and eluded in 20mM NaOAc, pH 6, 1 M NaCl. The GAGs were precipitated in 3 volumes on ethanol 24h at 4°C. The precipitate was collected at 9000g for 30min and dried at 50°C. The dried sample was then resuspended in water and desalted using a PD-10 column (GE Healthcare). The sample was then treated with hyaluronidase from *Streptomyces hyalurolyticus* (Seikagaku Biobusiness Corporation, Japan), and A mixture of *Flavobacterium heparinum* heparin lyase I, II, and III in succession. The chondroitin sulfate was then precipitated in three volumes of ethanol, dried, and resuspended in water. The structure, purity and concentration was determined by MS.

### 2.7 Biotinylation of the pl-CS

Purified CS from the placenta was modified with an activated biotin reagent (sulfo-NHS-LC-biotin, Thermo-Pierce, Rockford, IL) at the amine group of the residual peptide left at the reducing end of CS chain after the trypsin digestion purification step. In Short the placental CS-peptide (100μg) in 50μL of 50mM phosphate buffer, pH 7.4, was mixed with the biotin reagent (1.0μmol) and incubated at room temperature for 2h. 5μL of 1M ethanolamine was then added to the mix and incubated at room temperature for 20min to terminate the reaction. The biotin-modified placental CS was purified on a Superose 12 HR 10/300 column (GE Healthcare, Piscataway, NJ) using 0.2M ammonium acetate as the eluent. The eluted product was finally freeze-dried three times. The placental CS-Biotin powder was resuspended in H_2_O prior to use in experiments.

For immobilization of the purified biotinylated pl-CS on Biotin Chips a biotin chip (Attana AB) was allowed to stabilize in 10mM HEPES, 150mM NaCl, 0.005% Tween-20 at a flow rate of 100μL/min on the Attana Cell™ 200 instrument. The flow rate was lowered to 20μL/min and 100μg/mL streptavidin (Attana AB) was injected and allowed to interact with the surface. The pl-CS-biotin was then diluted to 50μg/ml and injected on the surface for immobilization.

### 2.8 Kinetic analysis

The chips were inserted in the Attana Cell 200 instrument (Attana AB) and allowed to stabilize in running buffer at 25μL/min (10μL/min for the AR experiments). The purified pl-CS experiment and tests of monoclonal antibodies were run in PBS. rVAR2 cell and tissue experiments were run in PBS containing Synblock diluted to 0.1 × stock solution (Immunochemistry Technologies). Once stable, the baseline was verified with repeated injections of running buffer. The analyte to be tested was dissolved in the appropriate running buffer and diluted two-fold to yield the given concentration range. All injections were performed by the C-Fast autosampler (Attana AB). Baseline was checked between sample injections, by injections with running buffer. Blank injections were subtracted from the sample injections in the final analysis. The analytes were tested for reactivity against blank surfaces and against the given negative controls. The data was prepared using the Attana Attache software (Attana AB) and curve fitting was performed in the TraceDrawer software (Ridgeview Instruments) using the fit models given in Table 1. For some experiments a low level of background binding was accounted for by fitting a 1:2 model. Only the true high affinity values (K_D_ values) are listed in Table 1. For CSA inhibition, rVAR2 at a concentration of 100nM was pre-incubated with 400μg/mL bovine CSA (Sigma) and injected over the surface. For the verification of tissue adherence, rVAR2 was injected over the tissue surface until saturation. The lid on the COP-1 Cartridge was removed and the tissue incubated 30min with *anti*-V5-FITC (Invitrogen) at RT. The chip was washed and viewed under a top-down fluorescence microscope.

### 2.9 Immunofluorescence

The given cancer cell lines were seeded at a subconfluent concentration on glass slides and allowed to adhere at 37°C, 5% CO_2_. The cells were then fixed in 4% PFA and washed in PBS. The cells were blocked for 1h at RT in 1% BSA, 5% FBS in PBS. The cells were then stained with V5 tagged rVAR2 or *anti*-AR (N-20, Santa Cruz) for 1h at RT in PBS containing 0.25% BSA. The cells were washed and incubated with *anti*-V5-FITC or anti-mouse-FITC in PBS containing 0.25% BSA. The slides were stained with DAPI, mounted, and viewed using confocal microscopy.

For the measurement of fluorescence intensity in the Attana chip rVAR2 detection on the prostate and tonsil tissues, 7 distinct areas of the tissue were selected and quantificated using the ImageJ software. Data is presented as Corrected Total Tissue Fluorescence (CTTF), for which the following formula was used, as described before: CTTF = Integrated density − (Area of selected region × Mean fluorescence of the background) [2], [20]. Quantification shown represents one measured experiment.

### 2.10 Microscospy

A Nikon *C*1 confocal microscope with a 60 × oil objective was used for imaging the IF cell stainings. A total of 5 representative pictures were taken per sample. Stainings were repeated twice. For analysis of the integrity of the immobilized tissue, a Leica DMLB with a Rolera-rx Fast 1394 camera and a 20 × objective was used.

## 3 Results

### 3.1 Preparation of FFPE tissue specimens on QCM biosensor chips

**Fig. 1:**
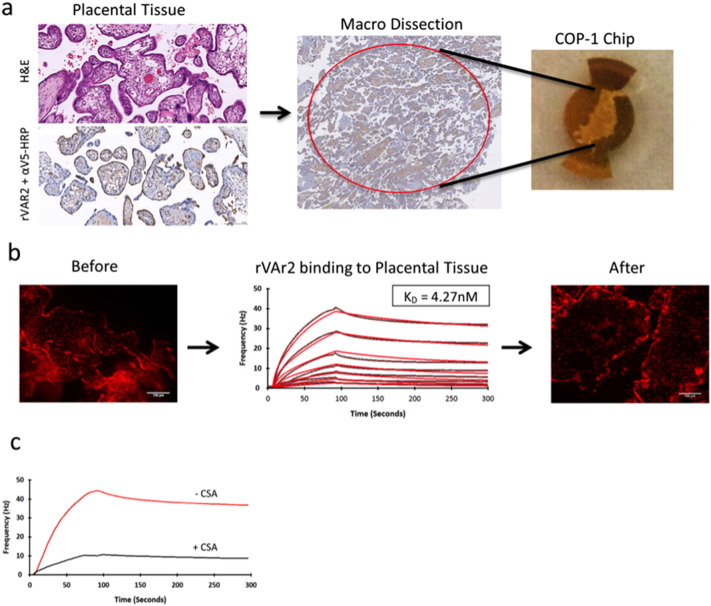
Tissue immobilization, treatment, and initial test of rVAR2 binding. A) placental tissue tested positive for the presence of pl-CS using rVAR2 in IHC was cut and immobilized on a COP-1 chip. Both H&E staining and rVAR2 staining of matched tissue is shown B) Following tissue immobilization and treatment the placental tissue was tested for the interaction with rVAR2 (1:1 dilution 200nM–3.125 nM) in an Attana Cell 200 instrument. Curve fitting was performed in TraceDrawer. Black curve is original data, red is fitted data. The K_D_ value is listed. Tissue integrity was visualized with nuclear ethidium bromide staining before and after the experiment. C) rVAR2 was injected over an immobilized piece of placental tissue with and without pre-incubation with 400μg/mL soluble bovine CSA.

Chips for the Attana QCM biosensor come with a wide variety of surfaces for the immobilization of different ligands to be analysed. Traditionally the kinetics of biomolecule interactions was studied with purified ligand immobilized on polystyrene or covalently linked through primary amines to reactive surfaces [22]. Recently, cells were grown on polystyrene chips optimized for cellular adherence (COP-1) and either fixed to the surface using PFA [8], [23] or tested as live cells [16]. Our aim was to immobilize a piece of FFPE primary human tissue to the chip for subsequent binding analysis. The cell COP-1 chips are plasma-treated to become hydrophilic, which is optimal for cellular adherence. We macro-dissected a small circular piece of placental tissue, at a thickness of 5 μm and placed it in the middle of a COP-1 surface that was covered with Poly-l-Lysine prior to tissue immobilization (Fig. 1a).

The detection of protein targets in FFPE tissue requires removal of the paraffin matrix and often requires further treatment for the regeneration and availability of the target antigens. We performed deparaffinization in EZprep solution and antigen retrieval in 10mM sodium citrate, 0.05% tween, pH 6 [27]. The chips were visually inspected at each step to ensure the presence of the tissue on the chip.

To test the availability of the pl-CS in the immobilized placental tissue we performed a binding experiment using a titration of rVAR2 protein (Fig. 1b). The integrity of the tissue was confirmed by visualizing the tissue with ethidium bromide DNA staining, before and after the binding experiment. rVAR2 highly interacted with the tissue with a K_D_ value of 4.3 nM. Furthermore, the interaction was CSA specific, as shown by the efficacy of inhibiting rVAR2 adhesion to the placental tissue with soluble CSA (Fig. 1c).

### 3.2 Affinity of the rVAR2:pl-CS interaction in primary tissue

**Fig. 2:**
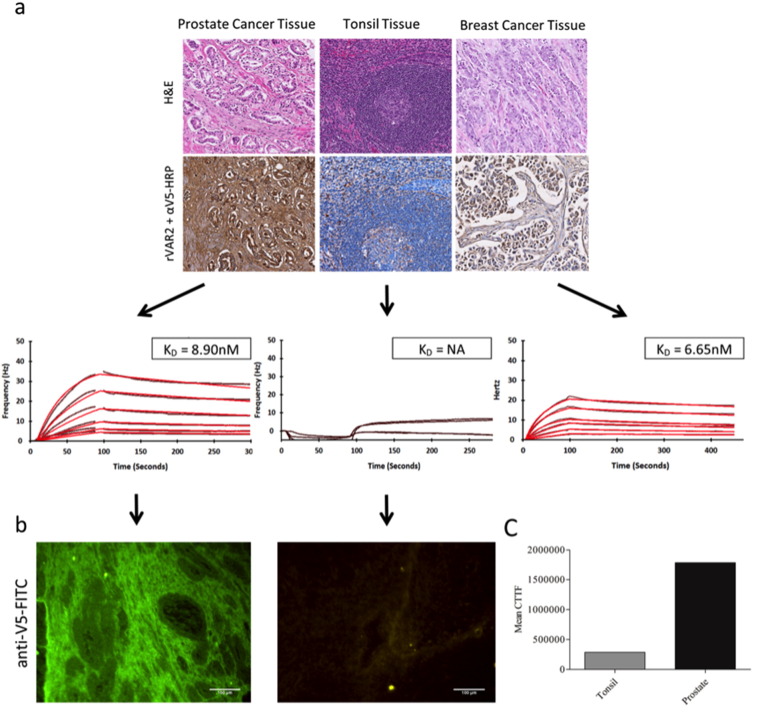
Investigating the interaction between rVAR2 and pl-CS in cancer Tissue. A) Prostate cancer, breast cancer, and tonsil tissue were stained with rVAR2 in IHC. Both H&E staining and rVAR2 staining of matched tissue is shown. Matched tissue was immobilized on COP-1 surfaces and subjected to binding analysis with rVAR2 (1:1 dilution 200 nM–3.125 nM) in an Attana Cell 200 instrument. Curve fitting was performed in TraceDrawer. Black curve is original data, red is fitted data. The K_D_ value is listed. B) To further test the interaction a piece of prostate cancer or tonsil tissue, immobilized on a COP-1 chip, were saturated with rVAR2 in an Attana Cell 200 instrument. The chips were disassembled and the presence of v5 tagged rVAR2 was visualized with *anti*-V5-FITC. C) Quantification of fluorescence from 10 pictures taken of the chips in B).

We have shown that rVAR2 specifically interacts with pl-CS in the placenta and in most cancers, without binding CS on normal tissue [27]. This observation was mainly based on staining FFPE tissues with rVAR2 in IHC. We wanted to study the kinetics of the interactions and the relative abundance of pl-CS in patient-derived FFPE primary cancer tissue. For this purpose, we immobilized FFPE tissue from a prostate cancer and a breast cancer patient on COP-1 chips, and performed binding analysis with a titration of rVAR2 (Fig. 2a). A piece of tonsil tissue was used as the control for normal tissue. rVAR2 interacted to a high degree with both the prostate and breast cancer tissue with comparable K_D_ values (8.9 nM and 6.7 nM, respectively). As previously observed [27], rVAR2 staining of the tonsil tissue in IHC was very weak, with only a limited amount of focal staining (Fig. 2a). In accordance with this rVAR2 did not interact with the tonsil tissue immobilized on the chip, in real time. To confirm these results and verify that rVAR2 interacted with the tissue, we disassembled the prostate cancer and tonsil chips following an experiment and confirmed the presence and localization of rVAR2 binding using an *anti*-V5-FITC antibody (Fig. 2b and c). This showed an intense staining of the prostate cancer tissue with very limited signal seen on the tonsil chip.

**Fig. 3:**
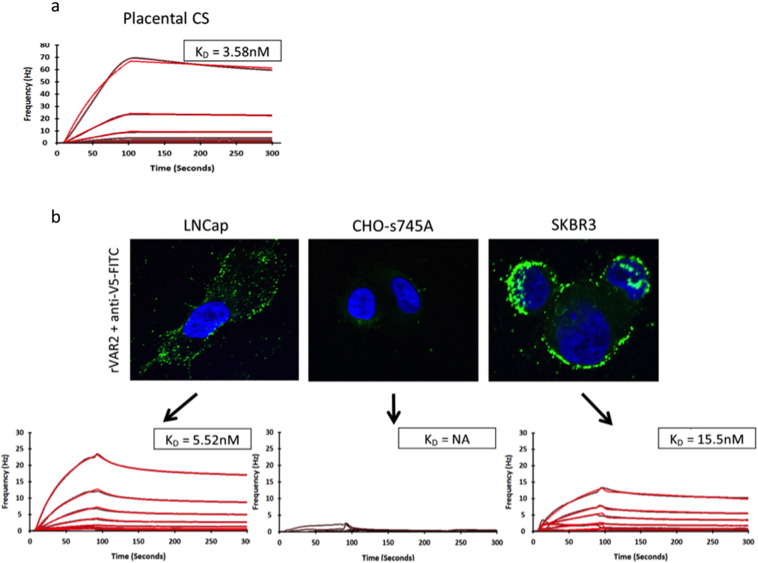
Testing the interaction of rVAR2 with purified pl-CS and fixed cancer cell lines. A) pl-CS was purified from human placental tissue, biotinylated, and immobilized on an Attana biotin chip, and subjected to binding analysis with rVAR2 (1:1 dilution 200 nM–3.125 nM) in an Attana Cell 200 instrument. Curve fitting was performed in TraceDrawer. Black curve is original data, red is fitted data. The K_D_ value is listed. B) LNCap, SKBR3 and CHO-A745 cells were stained for pl-CS using V5 tagged rVAR2 in IF. The cells were then fixed on Attana COP-1 chips and subjected to binding analysis with rVAR2 (1:1 dilution 200 nM–3.125 nM) in an Attana Cell 200 instrument. Curve fitting was performed in TraceDrawer. Black curve is original data, red is fitted data. The K_D_ value is listed.

To date, biosensor analysis has relied on the interaction between an analyte and an immobilized cell or purified receptor [5], [8], [16], [22], [23]. In line with this, we have previously investigated the interaction between several recombinant VAR2CSA proteins and immobilized bovine decorin [6], [7], as well as the interaction of rVAR2 with a melanoma cell [27]. To verify the interaction between rVAR2 and the immobilized tissue, we performed the same analysis on purified placental-like CS and cancer cells of prostate and breast cancer origin (Fig. 3). We purified pl-CS from placenta, biotinylated it through the peptide remaining from protease treatment, and immobilized it on an Attana biotin chip. As with the tissue, rVAR2 interacted highly with the pl-CS on the surface in the same concentration range and with a comparable K_D_ value (3.6nM) (Fig. 3a). To further verify the interaction we cultured an AR positive prostate cancer cell line (LNCap), and a breast cancer cell line (SKBR3) on the surface of a COP-1 chip, fixed them in PFA, and subjected them to the same analysis. Both cell lines were shown positive for the presence of pl-CS by rVAR2 staining in IF (Fig. 3b). The interaction analysis was comparable between the two cells lines and the analysis performed on the tissues and the purified pl-CS (Fig. 3b). A CHO cell selected for low xylotransferase activity (CHO-s745A) [9], and therefore low overall glycosaminoglycan (GAG) content, was used as the negative control for binding. This cell line was shown negative for the presence of pl-CS in IF, and was negative for the interaction with rVAR2 in the biosensor setup (Fig. 3b).

### 3.3 Affinity of an *anti*-AR antibody to prostate cancer tissue

**Fig. 4:**
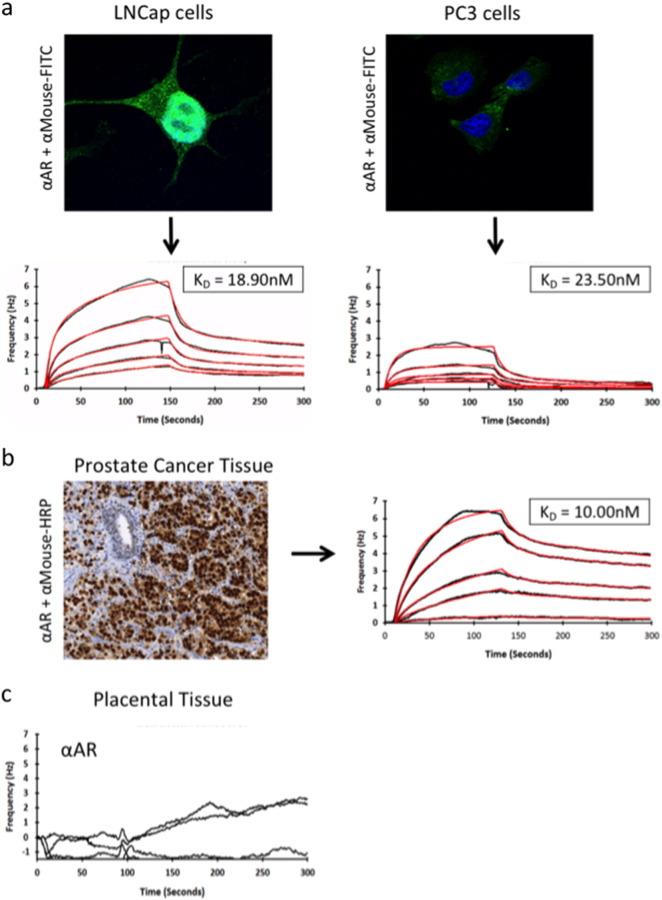
Investigating the Interaction between clinically relevant monoclonal antibodies and their targets in Cancer Tissue. A) LNCap and PC3 cells were tested for the expression of AR by IF. The cells were fixed to an Attana COP-1 surface. The chips were then subjected to binding analysis with anti-AR (N-20, Santa Cruz) (1:1 dilution 200 nM–3.125 nM) in an Attana Cell 200 instrument. Curve fitting was performed in TraceDrawer. Black curve is original data, red is fitted data. The K_D_ values are listed. B) A piece of primary prostate cancer tissue was selected for high AR expression in IHC, and a matched piece of tissue was immobilized on a COP-1 surface. Kinetic analysis was performed as in A. C) A piece of primary placental tissue was immobilized on a COP-1 surface and subjected to kinetic analysis as in A. Curve fitting was not possible due to no binding.

To further extend the validation of the tissue biosensor setup, we selected a well-characterized cancer marker for which a validated monoclonal antibody is available. We analysed the interaction of the *anti*-Androgen Receptor (AR) N-20 antibody, used in the characterization of prostate cancer [13], with endogenous AR in cancer cells and in FFPE prostate cancer tissue. LNCap cells are known to express high levels of AR [21]. In accordance with this the LNCap cells was stained by anti-AR in IF and the anti-AR antibody interacted with the LNCap cells immobilized on a COP-1 surface, with a K_D_ value of 18.9 nM (Fig. 4a). Contrary to this PC3 cells, which are known to be androgen independent [32], showed very little AR staining in IF and a corresponding low level of interaction with the AR-antibody when immobilized on the biosensor chip (Fig. 4a). Having shown that the antibody worked in the biosensor setup, we selected an AR positive FFPE primary prostate cancer tissue biopsy, immobilized it on a COP-1 surface, and performed binding analysis on the Attana Cell 200. As with the rVAR2 analysis, interaction to the tissue was comparable to that of the immobilized AR positive cells (K_D_ value of 10 nM) (Fig. 4b). A piece of AR negative placental tissue showed no interaction with the AR antibody (Fig. 4c), confirming the target specificity. When comparing the peak responses measured in Hz between rVAR2 and *anti*-AR binding to the primary tissues it is evident that rVAR2 binding is up to 10 fold higher than the with the antibody.

## 4 Discussion

Discovery and characterization of cancer-associated markers effective in cancer diagnosis and treatment is a main focus in modern medicine. Several targets have been described [13], [14]. Furthermore, we recently showed that the malarial VAR2CSA protein targets a specific type of placental-like chondroitin sulfate found in the placenta and on most cancer cells [27]. The field of medical science increasingly recognizes that a thorough molecular analysis of the biomarker recognition event is needed for full evaluation of a target for diagnostic and treatment purposes [11]. Such thorough analysis requires an in depth understanding of the molecular mechanism underlying the specific interaction as well as its kinetic properties. For this purpose scientists are utilizing a wide range of techniques including nuclear magnetic resonance (NMR) [12], [26], mass-spectrometry (MS) [12], Enzyme-linked-immunosorbent-assay (ELISA), isothermal titration calorimetry (ITC) [15], and biosensor technology [1], [5], [19], [22], [24], [31]. Common for these techniques is a requirement for the isolation and purification or recombinant expression of the target molecule. While these methods are valuable in describing a given biomarker and how to target it effectively, biomarker extraction is laborious and may interrupt key features only present in the biomarker's, or target's, native environment.

Current molecular pathology relies on the intensity and pattern of expression of the given biomarker using two-dimensional histopathological and IHC analysis. This work aims at adding an extra dimension of interaction time to classic pathology by performing kinetics of binding analysis directly on FFPE primary tissue specimens. We believe that this provides pathologists with a tool to evaluate disease specific targets not only by their presence and specific localization, but also by their molecular characteristics and how this relates to its direct interaction with a targeting molecule.

We macro-dissected out a small circular piece of paraffin-embedded tissue and put it on COP-1 Attana QCM chip surfaces. We then removed the paraffin and performed antigen retrieval. One problem was that the tissue did not stick sufficiently to non-treated COP-1 surfaces. A coating with poly-l-lysine was needed for proper immobilization. The antigen retrieval needed was optimized by IHC staining using the analytes in question. Subsequently, the tissue-coated chips were treated in an identical way, allowing for direct comparison. Since no light can go through the gold COP-1 surface, we visualized the immobilized tissue by a DNA stain and a fluorescent microscope before and after the experiments to ensure the integrity of the tissue. No change in morphology was seen, showing that the proposed method maintains proper tissue integrity for subsequent analysis. The exact epitope of recognition of the *anti*-AR (N-20) antibody and its molecular nature is not known. It is likely that antibody specific optimization on tissue treatments is needed to accommodate analysis of other antibodies, such as antibodies targeting conformational epitopes.

Having optimized the immobilization of the FFPE tissue, we performed binding experiments using rVAR2 and *Anti*-AR (N-20) monoclonal antibodies on tissues verified for the presence of the target antigen in question. All binding experiments were verified against appropriate cell lines, and in the case of rVAR2 also purified pl-CS. rVAR2 showed similar binding kinetics to purified placental-like CS, cell lines, and tissue. This confirms our strategy and supports the use of primary tissue for QCM experiments. It is common to evaluate specific binding in biosensor experiments by subtracting binding to a blank reference [25]. However, in working with immobilized cells and tissues we found that binding of an analyte to a blank chip is not representative of the background seen in binding to a chip coated with cells or tissues. When investigating the interaction between an analyte and a purified receptor in classic SPR, the surface area available for non-specific interactions on the chip can be considered comparable. This is not the case when investigating a chip covered in tissue or cells. Subtracting the signal from the reference chip therefore introduced a negative bias on the ‘true’ interaction, visualized as a negative spike in the real-time observation of the on-rate (Data not shown). Instead we selected cells and tissues negative for the ligand in question as proper negative controls, and presented them as separate figures to demonstrate the background independently. In this, rVAR2 did not interact with normal tissue (tonsil), confirming our previous observations that rVAR2 staining is placenta and cancer specific [27]. Furthermore, rVAR2 did not interact with the CS negative CHO-A475a cells and binding could be inhibited with soluble CSA, confirming the CS specificity. The analysis of the AR interaction showed the same correspondence with AR expression in both cell line and tissue setups. It is notable that such analysis was possible using a nuclear target.

Interestingly the data shows that pl-CS is abundantly present in tumour tissue compared to AR. This is evident from the high difference in max binding responses, with the Bmax response for rVAR2 being up to 10 times higher. This difference is not obvious in the IF staining and IHC which with the inherent enzymatic horseradish peroxidase staining is difficult to accurately quantify. The concept of target quantity is interesting for cancer therapy as targeting an abundant target may offer a more efficient *anti*-cancer effect.

We demonstrate that this novel method can be broadly used to determine the relative presence of a cancer specific target as well as the affinity of the targeting reagent for its receptor. Implementation of the method has the potential to inform cancer diagnostics as well as provide an important tool for screening of high affinity targeting reagents in drug development, and aid in patient stratification, companion diagnostics and dosage determination during therapeutic treatment.

## Contributions

T.M.C, A.S., M.D., and T.G.T., designed the research; T.M.C, M.A.P., H.Z.O., Y.M., N.S., M.R., and J.L., performed the experiments; L.F., provided useful reagents and helpful discussions; T.M.C., A.S, and M.D. wrote the manuscript.

## Conflicts of interest

The authors declare no conflicts of interest.

## Figures and Tables

**Table 1:** Curve fitting data for all QCM experiments. Listed is the tested analyte, the immobilized ligand, the model used for curve fitting in TraceDrawer, the obtained k_a,_ k_d_, and K_D_ values, and the estimated error in that particular fit. NA depicts experiments for which no kinetic fit could be obtained due to no analyte binding. These are representative experiments of repeated setups.

	Sample	Fit model	K_a_ (1/M ∗ s)	K_d_ (1/s)	K_D_ (nM)	Est. Error (nM)
rVAR2	Placental tissue	1:2	9.2 × 10^4^	3.9 × 10^− 4^	4.3	± 0.01
	Prostate cancer tissue	1:1	12.7 × 10^4^	11.3 × 10^− 4^	8.9	± 0.06
	Tonsil tissue	NA	NA	NA	NA	NA
	Breast cancer tissue	1:2	7.9 × 10^4^	5.3 × 10^− 4^	6.7	± 0.3
	Purified placental CS	1:1	12.3 × 10^4^	4.42 × 10^− 4^	3.6	0.05
	LNCap cells	1:2	9.4 × 10^4^	5.2 × 10^− 4^	5.5	± 0.0004
	SKBR3 cells	1:2	6.8 × 10^4^	10.5 × 10^− 4^	15.6	± 0.2
	CHO-s745A cells	NA	NA	NA	NA	NA
αAR	Prostate cancer tissue	1:2	13.0 × 10^4^	13.1 × 10^− 4^	10	± 0.04
	Placental tissue	NA	NA	NA	NA	NA
	LNCap cells	1:2	7.4 × 10^4^	14.0 × 10^− 4^	18.9	± 0.07
	PC3 cells	1:2	23.7 × 10^4^	55.5 × 10^− 4^	23.5	± 0.6
